# Oral Health and Oral Health-Related Quality of Life in Professional Soccer Players in Southern Italy: A Cross-Sectional Study

**DOI:** 10.3290/j.ohpd.c_1859

**Published:** 2025-02-18

**Authors:** Silvia Angelillo, Martina Ferrillo, Delfina Pacifico, Saverio Mirarchi, Leonzio Fortunato, Carmelo Nobile

**Affiliations:** a Silvia Angelillo Researcher, Department of Health Sciences, University of Catanzaro ‘Magna Graecia’, Catanzaro, Italy. Investigation, data curation, writing (original draft preparation), read and agreed to the published version of the manuscript.; b Martina Ferrillo PhDs, Department of Health Sciences, University of Catanzaro ‘Magna Graecia’, Catanzaro, Italy. Investigation, data curation, writing (original draft preparation), read and agreed to the published version of the manuscript.; c Delfina Pacifico Dentist, Department of Health Sciences, University of Catanzaro ‘Magna Graecia’, Catanzaro, Italy. Investigation, data curation, writing (original draft preparation), read and agreed to the published version of the manuscript.; d Saverio Mirarchi President, Calabria Regional Committee, Italian Football Federation (Federazione Italiana Giuoco Calcio – FIGC), Italy. Investigation, read and agreed to the published version of the manuscript.; e Leonzio Fortunato Associate Professor in Dentistry, Department of Health Sciences, University of Catanzaro ‘Magna Graecia’, Catanzaro, Italy. Methodology, visualisation, read and agreed to the published version of the manuscript.; f Carmelo Nobile Professor of Hygiene, Department of Pharmacy, Health and Nutritional Sciences, University of Calabria, Arcavacata of Rende, Cosenza, Italy. Conceptualisation, methodology, formal analysis, writing (review and editing), supervision, read and agreed to the published version of the manuscript.

**Keywords:** epidemiology, oral health, caries, periodontal disease, soccer players, sports dentistry

## Abstract

**Objectives:**

To evaluate the oral health of professional footballers and to investigate possible determinants of oral health as well as the self-reported impacts on well-being, quality of life (QoL), and performance.

**Methods:**

This cross-sectional study was carried out on professional soccer players of the Calabria region, Italy. The outcome measures were the following: DMFT (decayed, missing and filled permanent teeth), DMFS (decayed, missing and filled permanent teeth surfaces), BEWE (basic erosive wear examination), CPI (community periodontal index), CPITN (community periodontal index of treatment needs), Oral Health Impact Profile-14 (OHIP-14).

**Results:**

One hundred and sixty footballers were recruited from seven clubs. The median age of the players was 25 years (19–39) years. The mean DMFT was 2.8 ± 2.9, and the multiple logistic regression analyses showed a positive association with frequent intake of drinks rich in sugar (OR = 3.69, 95% CI = 1.59–8.56) and sports drinks (OR = 3.73, 95% CI = 1.09–12.75). Dental erosions were present in 48.1% of footballers and periodontal diseases in 50%, with a positive association with frequent intake of energy drinks (OR = 2.86, 95% CI = 1.09–7.51). The OHIP-14 showed that 30.6% of participants reported having had pain in their teeth/mouth/dentures occasionally.

**Conclusions:**

Results from the present study showed that the oral health of professional soccer players in Southern Italy was poor, especially regarding caries, erosion, and periodontal diseases. Moreover, OHIP-14 showed an impact on oral health in soccer players’ QoL, revealing that poor oral health negatively affected professional well-being and performance. Results suggest the need for prevention interventions for professional athletes.

In the last decades, several studies have been conducted to document the oral health status of professional athletes, and there is growing evidence on the relationship between poor oral health and negative self-reported impacts on well-being, training, and performance.^1,13,17,19,20^

Studies providing high-quality data on oral health and its impact on quality of life (QoL) are important to establish appropriate preventive and health promotion strategies, and self-reported measures help to understand the issues that affect athlete development and welfare.^20^

A previous study by Needleman et al^19^ was conducted to evaluate the oral conditions and the effect of oral health on well-being, training, and performance in athletes participating in the London 2012 Games. The authors recruited 300 athletes from 25 sports and reported high levels of preventable conditions, including periodontal disease (gingivitis 76% of athletes, periodontitis 15% of athletes), dental caries (55% of athletes), and dental erosion (45% of athletes), with 30% of participants reporting an impact of oral health on their QoL and almost 20% about their training or performance.

An interesting study by Gallagher et al^13^ analysed the oral health of 352 athletes from 11 sports and showed a high prevalence of oral diseases. More in detail, the oral assessment revealed caries in 49.1% of athletes, gingival bleeding on probing and calculus in 77.0%, and pocket probing depth of 4 mm or more in 21.0%. Moreover, 32.0% of athletes reported a negative oral health-related impact on sports performance.

A systematic review performed on professional and elite sports athletes showed similar results across a wide range of sports.^1^ These findings led to the development of position/consensus statements with recommendations to promote oral health and related research as part of sports and exercise medicine.

The first study performed among professional football players was conducted in the United Kingdom and evaluated the prevalence of teeth and periodontal tissue problems in a sample of 187 professional footballers enrolled on eight teams.^18^ The results showed poor oral health with: 37% of the players with active dental caries, 53% with dental erosion, and 5% with moderate-severe periodontal disease, with 20% reporting an impact on QoL and 7% on training or performance. This study provided strong evidence to support oral health screening in professional football players.

Oral health is a determinant of life quality, and there is a wealth of literature demonstrating that poor oral health, including caries, periodontal disease, and pericoronitis, may affect QoL.^1,13,16–18^ Indeed, other than symptomatic effects, psychosocial impacts of poor oral health are recognised as a major influence on QoL.^2^

Thus, previous studies suggested a relationship between poor oral health and self-reported negative impacts,^1,13,17,19,20^ but no studies were performed on professional football players from soccer teams in Southern Italy.

We hypothesised that oral disease might have an impact on general health, with consequent negative sports performance impact due to difficulties with eating, sleeping and socialisation.

Therefore, the aim of this study was to evaluate the oral health of professional football players and to investigate possible determinants of oral health as well as the self-reported impacts on well-being, performance, and QoL.

## MATERIALS AND METHODS

This cross-sectional study was carried out on professional football players of the Calabria Region, Italy. All Calabrian soccer teams (F.C. Crotone S.r.l., Catanzaro Calcio 2011 S.r.l., Cosenza Calcio S.r.l., Urbs Sportiva Reggina S.r.l., U.S. Vibonese Calcio) were offered the opportunity for their athletes to participate and those that gave consent were included in the study. A letter summarising the purpose of the study and pointing out the voluntary and confidential nature of participation, an informed consent form, and a questionnaire were given to each selected player. Each participant gave written consent prior to the start of the study. Participants were also told that the information they provided would have been reported only in aggregate form. Eligible athletes were professional football players, aged ≥ 18 years, able to understand the consent process and the provided questionnaire.

The present study was conducted according to the STrengthening the Reporting of OBservational studies in Epidemiology (STROBE) Guidelines^26^ and the study protocol was approved by the Ethical Committee of Calabria Region (protocol number: 351/2016).

A survey composed of the following five sections was administered: (1) demographics and socioeconomic status (age, marital status, highest level of education obtained); (2) oral hygiene practices, utilisation of dental healthcare services; (3) sweet and energy drinks intake frequency; and (4) impact of oral health on QoL.

The frequency of toothbrushing was scored on a three-point scale, ranging from never or less than once a day to more than once a day.

Participants were asked to describe their utilisation of dental services in the previous year. The questions included information on dental problems, number of visits, and main reason for each visit and received treatment. They were defined as having a regular pattern of dental attendance if they claimed to have a dental visit at least once a year; otherwise, dental attendance was defined as ‘irregular’.

Consumption of sweets was scored on a four-point scale, ranging from never or less than once a week to once a day.

The oral health-related quality of life (OHRQoL) was assessed using the Oral Health Impact Profile-14 (OHIP-14). OHIP is a questionnaire that measures people’s perception of the social impact of oral disorders on their well-being. Slade^23^ in 1997 developed a short-form of it with 14 questions, named OHIP-14, which showed good reliability, validity, and precision. Fourteen items of OHIP are divided into seven dimensions: functional limitation, physical discomfort, psychological discomfort, physical disability, psychological disability, social disability, and handicaps.

Following the interview, the same dental clinician with extensive clinical experience, who had been trained and calibrated prior to the commencement of the study, assessed the oral health conditions of each subject by performing all clinical measurements in a regular dental room, according to previous studies.^21,22^ Dental clinical examinations were performed under artificial light by means of a plane mouth mirror, explorer, and a periodontal ball-pointed probe recommended by the World Health Organization (WHO), for the detection of the following outcomes: DMFT (decayed, missing and filled permanent teeth), DMFS (decayed, missing and filled permanent teeth surfaces), BEWE (basic erosive wear examination), CPI (community periodontal index), and CPITN (community periodontal index of treatment needs).

The DMFT index is one of the most common methods in oral epidemiology for assessing dental caries. DMFT is applied to permanent dentition and is expressed as the total number of teeth that have decayed (D), missing (M) or filled (F) in an individual. The score for each individual tooth is equal to 1. The score per individual can range from 0 to 28, because third molars are not included in the scoring.

The DMFS index is another method in oral epidemiology for assessing dental caries. This index counts each affected surface, yielding decayed, missing, and filled surfaces (five per posterior tooth and four per anterior tooth). The score per individual can range from 0 to 128, because third molars are not included in the scoring.

The BEWE is a tool that evaluates dental erosion.^3^ Dental erosion is the loss of hard dental tissue caused by the chemical dissolution of the mineral component, not caused by microorganisms. Erosion can, therefore, also affect those with excellent oral hygiene. The main cause of tooth erosion is contact with acidic substances, such as in gastric acid reflux disease. The BEWE is a partial scoring system recording the most severely affected surface in a sextant and the cumulative score guides the management of the condition for the practitioner. The four-level score grades the appearance or severity of wear on the teeth from no surface loss (0), the initial loss of enamel surface texture (1), distinct defect, hard tissue loss (dentine) less than 50% of the surface area (2), or hard tissue loss more than 50% of the surface area (3).

The CPI evaluates, according to a WHO recommendation, the severity and degree of periodontal diseases (gingivitis, periodontitis). Periodontal diseases are classified into five degrees according to their severity: healthy periodontal conditions (0); gingival bleeding on probing (1); calculus and bleeding (2); periodontal pocket 4–5 mm (3); periodontal pocket ≥ 6 mm (4).

Periodontal health status and treatment needs were assessed using the criteria of the CPITN as recommended by the WHO. CPITN measures the following conditions: no treatment needs (score 0), needs to implement oral hygiene (score 1), needs a professional cleaning of the teeth, with an increase in their oral hygiene (score 2), in addition to the above measures, more severe periodontal interventions (score 3–4).

Total and partial edentulousness, the presence of various types of dentures, and the prosthetic treatment need were recorded separately for both jaws, following the procedures of the WHO. Each jaw was examined and classified into one of five categories: no prostheses, one bridge, two or more bridges, partial denture, bridge and partial denture, or complete denture. Only subjects who were wearing or could show their dentures at the examination were recorded as denture-wearers.

Statistical analysis was developed using STATA software program, version 16 (Stata Corporation. College Station, Tx).

Data were summarised using frequencies and percentages for categorical data and mean and standard deviations for continuous data. T-test, Chi-square test, Chi-square for trend, and Fisher exact test were used to examine the potential association between the outcomes of interest and the independent variables.

Stepwise multiple logistic regression analyses were performed to determine the independent association of explanatory variables with the following outcomes of interest: DMFT (Model 1), DMFS (Model 2), BEWE (Model 3), and CPI (Model 4).

In Model 1, players were divided into those who had a DMFT = 0, versus all others; in Model 2, football players were divided into those who had a DMFS = 0, versus all others; in Model 3, athletes were divided into those who had a BEWE = 0, versus all others; in Model 4, football players were divided into those who had a CPI = 0, versus all others.

The following explanatory variables were included in all models: age, marital status, highest level of education obtained, oral hygiene practices, frequency of intake of foods and drinks rich in sugar, frequency of intake of sport drinks and energy drinks, smoking status, dental problems, and use of dental healthcare services in the past year.

A backward elimination procedure was applied by setting at P value > 0.4 the significance level for dropping variables from the models. A P value less than 0.05 was considered statistically significant. Adjusted odds ratio and 95% confidence intervals were calculated.

## RESULTS

One hundred and sixty footballers were recruited from seven clubs (F.C. Crotone S.r.l., Catanzaro Calcio 2011 S.r.l., Cosenza Calcio S.r.l., Urbs Sportiva Reggina S.r.l., U.S. Vibonese Calcio) (see Table 1). Reasons for nonparticipation were less than 18 years of age and withholding of consent. The median age of the players was 25 years (range 19–39 years). A large majority of athletes (90%) reported being single and only 15% of them reported having a university degree. Almost two-fifths of players (38.1%) reported having had dental problems within the past 12 months, and two-thirds (66.9%) reported having been to the dentist in the past 12 months. More than four-fifths of players (83.7%) reported eating sugar-rich foods at least once a day. Consumption of sports drinks or energy drinks at least once a day was reported by 61.3% of players. Almost a fifth of players (18.1%) reported current tobacco use, mostly cigarettes.

**Table 1:** Oral health status of the included athletes (n = 160)

Healthy teeth	Median	Range
Overall athletes n = 160	26	15–28
Number of athletes (n) with caries	n	%
n = 160	60	37.5
Number of athletes (n) with caries by team (n = 160)	n	%
A = 22	12	54.5
B = 23	7	30.4
C = 18	6	33.3
D = 24	10	41.7
E = 26	4	15.4
F = 25	12	48
G = 22	9	40.9
Number of athletes (n) with fillings or with teeth to be filled	n	%
n = 160	115	71.9
Number of athletes (n) with fillings or with teeth to be filled by club	n	%
A = 22	18	81.8
B = 23	19	82.6
C = 18	12	66.7
D = 24	19	79.2
E = 26	14	53.8
F = 25	19	76
G = 22	14	63.6
Number of athletes (n) with DMFT (yes)	n	%
n = 160	114	71.3
Number of athletes (n) with DMFT (yes) by club	n	%
A = 22	18	81.8
B = 23	19	82.6
C = 18	12	66.7
D = 24	20	83.3
E = 26	13	50
F = 25	19	76
G = 22	13	59.1
DMFT by club	Median	Range
A = 22	2.5	0–13
B = 23	2	0–11
C = 18	1	0–12
D = 24	3.5	0–10
E = 26	0.5	0–11
F = 25	2	0–7
G = 22	2	0–7
Number of athletes (n) with DMFS (yes)	n	%
n = 160	117	73.1
Number of athletes (n) with DMFS (yes) by club	n	%
A = 22	18	81.8
B = 23	19	82.6
C = 18	12	66.7
D = 24	20	83.3
E = 26	13	50
F = 25	20	80
G = 22	15	68.2
DMFS by club	Median	Range
A = 22	4	0–21
B = 23	3	0–36
C = 18	1.5	0–17
D = 24	4.5	0–30
E = 26	0.5	0–36
F = 25	3	0–13
G = 22	2	0–12

The median number of healthy teeth per athlete (excluding third molars from the calculation) was 26 (range 15–28), with 37.5% having at least one tooth affected by dental caries, 21.3% having one or more missing teeth, and 48.8% with one or more restorations. In footballers with one or more restorations, the mean number of teeth with restorations was three. When combining caries, missing teeth, and restorations together (DMFT), 71.3% of all footballers had at least one decayed, missing, or restored tooth (mean DMFT = 2.8, SD ± 2.9) (see Table 1 for further details).

Dental erosion, assessed with the BEWE scoring system, was present in 48.1% of footballers (Table 2). In those with erosion, 84.4% had an initial loss of enamel surface texture (grade 1), 13% had a distinct defect, loss of hard tissue (dentin) less than 50% of the surface (grade 2), and 2.6% had a hard tissue loss more than 50% of the surface area (grade 3). In the model evaluating BEWE (Model 3, Table 5), there was no statistically significant association between dental erosion and frequency of intake of foods or drinks rich in sugar, or sports drink, or energy drink.

**Table 2:** Basic erosive wear examination (BEWE) in the included athletes (n = 160)

n = 160	77	48.1
Grade of BEWE	n	%
0	83	51.9
1	65	40.6
2	10	6.3
3	2	1.2

**Table 5:** Stepwise multiple logistic regression models for potential determinants of the different outcomes of interest

	Model 1	Model 2	Model 3	Model 4
DMFT	DMFS	BEWE	CPI
Log likelihood = –79.54; χ² = 32.89; P <0.0001	Log likelihood = –77.03; χ² = 32.18; P <0.0001	Log likelihood = –108.03; χ² = 5.53; P = 0.1369	Log likelihood = –104.76; χ² = 12.29; P = 0.0915
OR	OR	OR	OR
(95% CI)	(95% CI)	(95% CI)	(95% CI)
Age, continuous	1.14	1.12	1.06	1.05
	(1.03–1.26)	(1.01–1.24)	(0.99–1.14)	(0.97–1.14)
**Marital status**
Single*	^#^	^#^	^#^	1.00
Married	^#^	^#^	^#^	0.34
				(0.09–1.29)
**Highest level of education obtained**
High school diploma or lower qualification*	^#^	^#^	^#^	^#^
University degree	^#^	^#^	^#^	^#^
**Frequency of brushing teeth**
<2 per day*	^#^	^#^	1.00	^#^
>2 per day	^#^	^#^	0.63	^#^
			(0.33–1.21)	
**Use of mouthwash**				
No*	1.00	1.00	^#^	1.00
Yes	1.77	2.26	^#^	1.45
	(0.8–3.94)	(0.97–5.26)		(0.75–2.81)
**Frequency of intake of foods rich in sugar**
Never or rarely*	^#^	^#^	^#^	1.00
Often or every day	^#^	^#^	^#^	0.27
				(0.02–3.06)
**Frequency of intake of drinks rich in sugar**
Never or rarely*	1.00	1.00	^#^	1.00
Often or every day	3.69	3.38	^#^	0.6
	(1.59–8.56)	(1.43–7.99)		(0.3–1.19)
**Frequency of intake of sport drinks**
Never or rarely*	1.00	1.00	^#^	^#^
Often or every day	3.73	3.69	^#^	^#^
	(1.09–12.75)	(1.04–13.09)		
**Frequency of intake of energy drinks**
Never or rarely*	^#^	1.00	^#^	1.00
Often or every day	^#^	0.54	^#^	2.86
		(0.15–1.9)		(1.09–7.51)
**Smoking status**				
No*	^#^	^#^	^#^	1.00
Yes	^#^	^#^	^#^	0.63
				(0.26–1.51)
**Dental problems in the past year**
No*	1.00	1.00	^#^	^#^
Yes	3.57	4.16	^#^	^#^
	(1.48–8.61)	(1.65–10.49)		
**Use of dental healthcare services in the past** year
No*	^#^	^#^	1.00	^#^
Yes	^#^	^#^	0.72	^#^
			(0.36–1.43)	

Periodontal diseases (gingivitis and periodontitis), evaluated with CPI, affected 50% of players (Table 3). In those with periodontal diseases, 52.5% had gingival bleeding on probing (score 1), 35% had calculus and bleeding (score 2), 7.5% had a periodontal pocket between 4 and 5 mm (score 3) and 5% had a periodontal pocket ≥ 6 mm (score 4). The model evaluating CPI (Model 4, Table 5) showed a positive association with frequent intake of energy drinks (OR = 2.86, 95% CI = 1.09–7.51), while there were no statistically significant associations between frequency of intake of foods and drinks rich in sugar or sports drink and CPI.

**Table 3:** Community periodontal index (CPI) of the included athletes (n = 160)

n = 160	80	50
Degree of periodontal disease CPI	n	%
0	80	50
1	42	26.3
2	28	17.5
3	6	3.7
4	4	2.5
		

The CPITN assessment revealed that periodontal treatment was necessary in 50% of the participants (Table 4). More in detail, 26.3% needed to implement home oral hygiene, 17.5% needed a professional cleaning of the teeth, with an increase in their home oral hygiene, and 6.2% needed more severe periodontal interventions.

**Table 4:** Community periodontal index of treatment needs (CPITN) of the included athletes (n = 160)

n = 160	80	50
Necessary treatment CPITN	n	%
0	80	50
1	42	26.3
2	28	17.5
3	10	6.2

Results from the stepwise multiple logistic regression models were reported in Table 5. The model evaluating DMFT (Model 1, Table 5) showed that experience of caries, missing teeth or dental restorations increased with age (OR = 1.14, 95% CI = 1.03–1.26). Furthermore, the results of this model showed a positive association with frequent intake of drinks rich in sugar (OR = 3.69, 95% CI = 1.59–8.56) and sport drinks (OR = 3.73, 95% CI = 1.09–12.75). Dental problems in the past year were also positively associated with DMFT (OR = 3.57, 95% CI = 1.48–8.61). There were no statistically significant associations between DMFT and the frequency of intake of foods rich in sugar, or energy drinks, or a visit to the dentist in the past year.

The DMFS model (Model 2, Table 5) also demonstrated a significant association with increasing age (OR = 1.12, 95% CI = 1.01–1.24). Furthermore, frequent intake of drinks rich in sugar (OR = 3.38, 95% CI = 1.43–7.99) and sports drinks (OR = 3.69, 95% CI = 1.04–13.09) and dental problems in the past year were positively associated with DMFS (OR = 4.16, 95% CI = 1.65–10.49). There were no statistically significant associations between DMFT and the frequency of intake of foods rich in sugar, or energy drinks, or a visit to dentist in the past year.

The impact of oral health on quality of life is reported in Table 6.

**Table 6:** Impact of oral health on quality of life in the included athletes (n = 160)

Questions	Never/Rarely	Occasionally	Often/Very often
n	%	n	%	n	%
Have you had trouble speaking a few words due to tooth/mouth/denture problems?	151	94.4	6	3.7	3	1.9
Have you had a feeling that your sense of taste has worsened due to tooth/mouth/denture problems?	152	95	7	4.4	1	0.6
Have you had pain in your teeth/mouth/denture?	105	65.6	49	30.6	6	3.8
Have you felt uncomfortable eating certain foods due to tooth/mouth/denture problems?	124	77.5	31	19.4	5	3.1
Have you ever felt uncomfortable due to tooth/mouth/denture problems?	148	92.5	10	6.3	2	1.2
Have you ever felt tense due to tooth/mouth/denture problems?	148	92.5	12	7.5	0	0
Has your diet ever been unsatisfactory due to tooth/mouth/denture problems?	156	97.5	4	2.5	0	0
Have you ever stopped meals for tooth/mouth/denture problems?	147	91.9	10	6.2	3	1.9
Have you ever found it difficult to relax due to tooth/mouth/denture problems?	139	86.9	16	10	5	3.1
Have you ever felt a little embarrassed with other people due to tooth/mouth/denture problems?	151	94.4	8	5	1	0.6
Have you ever felt a little irritated towards other people due to tooth/mouth/denture problems?	148	92.5	8	5	4	2.5
Have you ever had trouble doing your usual jobs due to tooth/mouth/denture problems?	157	98.1	3	1.9	0	0
Have you ever thought that your life, in general, was less satisfying for tooth/mouth/denture problems?	148	92.5	10	6.3	2	1.2
Have you ever been totally unable to perform certain functions for tooth/mouth/denture problems?	154	96.3	6	3.7	0	0

Overall, 30.6% of participants reported having had pain in their teeth/mouth/denture occasionally and 3.8% often or very often. Moreover, 19.4% of football players reported to have been felting uncomfortable eating certain foods due to tooth/mouth/denture problems occasionally, and 3.1% often or very often.

Furthermore, 10% of players reported to have had difficulty to relax due to tooth/mouth/denture problems occasionally and 3.1% often or very often.

Over 90% of the footballers did not report a negative impact of oral health on their quality of life answering the other questions of the OHIP-14 questionnaire.

## DISCUSSION

Oral health is part of general health, and is recognised as an essential component of QoL.^7,9,26^ Poor oral health negatively affects OHRQoL and could adversely affect athletic performance and training.^17,19,20^ To achieve the maximum level of fitness required of a soccer player, general and oral health problems, which may occur before or during competition, should be prevented.^25^ Dentistry applied to sports should focus on the prevention and treatment of orofacial diseases, in order to maintain and improve the oral health of athletes, disseminating information and new knowledge in the sports medicine community.

One hundred and sixty footballers were included in this study to evaluate the oral health of professional footballers and to investigate possible determinants of oral health as well as the self-reported impacts on well-being, QoL, and performance.

The DMFT was the most commonly used index that reflects oral and dental health in the community. In the present study, DMFT index showed that 71.3% of footballers had at least one decayed, missing or restored tooth, with a positive association with intake of drinks rich in sugar (OR = 3.69) and sports drinks (OR = 3.73). More in detail, the 37.5% of the athletes had at least one tooth affected by dental caries and 48.8% of them had one or more restorations. These data were in line with Needleman et al^18^ who showed a consumption of sports drink by 63.7% players of at least three times per week, and reported that 37% of the players had active dental caries. Poor oral health in athletes may be related to the fact that endurance sports potentially reduce salivation, increasing the risk of caries and erosion.^12^

A systematic review by Ashley et al^1^ investigated the epidemiology of oral disease and trauma in athletes and investigated the impact of oral health on sporting performance. The included studies on footballers had small sample sizes that ranged from 18 to 34, which were unlikely to be representative. Despite only limited data, the oral health was shown to be poor. Indeed, the mean DMFT values ranged from 5.7 to 8 and were higher than the mean DMFT of 2.8 ± 2.9 showed in the present study.

The 48.1% of footballers revealed dental erosion, assessed with the BEWE scoring system. No statistically significant association between dental erosion and frequency of intake of foods, or drinks rich in sugar, or sports drink, or energy drink. These results were in accordance with Needleman et al^18^ who reported dental erosion in 53.1% of athletes with no statistically significant association with sports drink frequency.

At the same time, periodontal disease is one of the more common inflammatory diseases in the adult population,^6,8^ thus making necessary an early diagnosis to avoid the progression of the disease.^28^ However, although early identification of periodontitis is essential to avoid further progression of the disease, even today, periodontal diseases are often diagnosed quite late. A recent systematic review by Ferreira et al^10^ revealed an association between a self-perceived decrease in sports performance and periodontal disease, and the authors suggested that athletes should undergo regular visits to the dentist and perform an adequate daily home hygiene including brushing and flossing.^11,23^

The CPI revealed that 50% of players were affected by periodontal diseases, with 52.5% reporting only bleeding on probing, 35% calculus and bleeding, 7.5% periodontal pocket between 4 and 5 mm, and 5% periodontal pocket ≥6 mm. A positive association with frequent intake of energy drinks was shown (OR = 2.86). Our results were slightly better than those reported by previous reports that found inflammatory gingivitis in 76–80% of individuals and irreversible periodontitis in 5–8.3%.^1,19^ However, these studies evaluated the periodontal status using the Basic Periodontal Examination, thus a direct comparison was not possible. On the other hand, the study by Gay-Escoda et al^14^ did not report periodontitis in soccer players using the gingival index and the probing pocket depth, probably because of the younger age of the included subjects (mean aged 21 ± 1.6 years).

Results from the OHIP-14 revealed that more than 30% of the subjects reported to have occasionally had pain in relation to teeth/mouth/denture, and that 10% occasionally found it difficult to relax due to tooth/mouth/denture problems. Thus, according to Ashley et al,^1^ results showed an impact of oral health on soccer player QoL. An interesting study by Gay-Escoda et al^14^ reported the impact of oral health on performance of 30 professional soccer players of the F.C. Barcelona and showed that 16.7% of athletes had experienced tooth pain during training or competition and 40% had suffered trauma directly to the temporomandibular joint.

Accordingly, Needleman et al^19^ evaluated the effect of oral health on well-being of 302 athletes from 25 sports and showed that more than 40% of them were disturbed by their oral health with 28% reporting an impact on QoL and 18% on training and performance.

Poor oral health in athletes is not a new finding. It should be taken into account that several factors may be involved, such as diet and use of sports drinks,^4,5^ exercise-induced immune suppression,^15^ and reduced salivary flow during exercise.^5^ However, the oral problems found in this study are preventable, especially considering the young age of the players.

It is highly plausible that oral health could affect performance in view of the well-recognised effects of oral health on health-related quality of life.^16^ The mechanisms could include pain, psychological impacts, and effects on eating and the possible negative impact from poor oral health on elite performance warrants further investigation, although this is not a new finding.

Results from the present study suggest the importance of oral examinations before the beginning of the soccer season, and periodical follow-ups by dentists, to prevent the occurrence of oral pathologies, integrating the oral health promotion within the overall athlete care.

The main strengths of this study are the high number of professional soccer players recruited (n = 160) and the comprehensiveness of the oral health assessment, which provided information on caries, erosions, periodontal status, and OHRQoL. Moreover, the examinations were carried out by the same dental clinician with extensive clinical experience, who had been trained and calibrated prior of the commencement of the study.

## CONCLUSION

Taken together, results from the present study showed that the oral health of professional soccer players in Southern Italy was poor, especially in terms of caries, erosion, and periodontal diseases. Moreover, a positive association between caries and periodontal disease with frequent intake of drinks rich in sugar and sport drinks was reported. Furthermore, OHIP-14 showed an impact of oral health on soccer player QoL, revealing that poor oral health negatively affected professional’s well-being.

As oral health is an important element of overall health, results from the present study suggest the need for oral examinations before the beginning of the soccer season, and periodical follow-ups by dentists, to prevent the occurrence of oral pathologies, integrating the oral health promotion within the overall athlete care.

### Data Availability Statement

The data that support the findings of this study are available from the corresponding author upon reasonable request.

### Funding Statement

This research received no external funding.

### Conflict of Interest

The authors declare that the research was conducted in the absence of any commercial or financial relationships that could be construed as a potential conflict of interest.

### Ethics Approval Statement

The study protocol was approved by the Ethical Committee of Calabria Region (protocol number: 351/2016).

### Patient Consent Statement

Each participant gave written consent prior to the start of the study.
